# Oxidation Kinetics, Morphology Evolution, and Formation Mechanisms of the High-Temperature Oxide Scale for Cr-Alloyed Automotive Beam Steels

**DOI:** 10.3390/ma18163774

**Published:** 2025-08-12

**Authors:** Jiang Chang, Yuantao Hu, Yonggang Yang, Chen Jiang, Jianling Liu, Borui Zhang, Xiong Yang, Zhenli Mi

**Affiliations:** 1 National Engineering Research Center for Advanced Rolling and Intelligent Manufacturing, University of Science and Technology Beijing, Beijing 100083, China; d202110582@xs.ustb.edu.cn (J.C.); m202321389@xs.ustb.edu.cn (Y.H.); mizhen_li@126.com (Z.M.); 2 Sinosteel Anhui Tianyuan Technology Co., Ltd., Ma’anshan 243004, China; 15061322853@163.com; 3 State Key Laboratory of Powder Metallurgy, Central South University, Changsha 410083, China; 4 Ningbo Iron & Steel Co., Ltd., Ningbo 315800, China; zhangborui@ningbosteel.com (B.Z.); yangxiong111000@126.com (X.Y.)

**Keywords:** high-temperature oxidation, oxidation mechanism, morphology characterization, automotive beam steels

## Abstract

The oxidation behaviors of varying Cr-alloyed automotive beam steels—0.015 wt.% Cr, 0.15 wt.% Cr, and 1 wt.% Cr—were investigated using isothermal oxidation experiments. The morphologies of the oxide scale were characterized, and the formation mechanisms were analyzed to understand the change in the oxidation kinetics of the investigated steels. The results show that a small amount of Cr, up to 0.15 wt.%, can reduce oxidation kinetics; the addition of Cr at 1 wt.% causes the oxidation rate to decline at a low isothermal temperature, but the hindrance effect expires when the oxidation temperature is above 1050 °C. The oxidation scale, including the inner FeO layer, the intermediate Fe_3_O_4_ layer, and the outer Fe_2_O_3_ layer, exhibits a morphological evolution from marble-like to pore-like, then whisker-like, flocculation-like, fine oxide grains, and finally coarse oxide grains. With increasing Cr addition, the thickness of the FeO layer decreases significantly, leading to a reduction in the total thickness of the oxidation scale. During the oxidation process of the investigated steel with 0.15 wt.% Cr, a Cr-rich layer and FeO-(Cr, Fe, Mn)_3_O_4_ eutectic form; meanwhile, FeO-(Cr, Fe)_2_O_3_ eutectic and Si-rich oxides, as well as a (Cr, Si)-rich layer, occur in the oxidation scale when 1 wt.% Cr is added to the steel. The occurrence of voids in the (Cr, Si)-rich layer is responsible for the increasing oxidation kinetics of the 1 wt.% Cr steel when the isothermal temperature is above 1050 °C, and the optimal Cr concentration in automotive beam steel is 0.15 wt.%, considering both oxidation resistance and cost.

## 1. Introduction

With the aim of reducing its carbon footprint, the automotive industry is increasingly applying advanced steels, for example, high-strength beam steels, in the body of automobiles [1,2,3]. However, significant reoxidation occurs during heating and hot rolling processing at 1000–1250 °C, generating multilayer iron oxides (FeO, Fe_2_O_3_, Fe_3_O_4_) that account for 0.5–3.0% material loss and impair surface quality [4,5]. Given the direct correlation between oxide scale characteristics and final product quality, enhanced high-temperature oxidation resistance offers multiple benefits: (i) reduced scale formation, (ii) improved material yield, and (iii) lower surface treatment expenses. Therefore, high oxidation resistance is a vital capacity for automotive beam steels that would be beneficial for their use in manufacturing structural components.

Investigations [6,7,8], which examine the oxidation scale on pure iron and low-carbon steels, show that a classical three-layer structure, namely FeO + Fe_3_O_4_ + Fe_2_O_3_, forms during the oxidation process. Moreover, the inner FeO layer, the intermediate Fe_3_O_4_ layer, and the outer Fe_2_O_3_ layer have a thickness ratio of 95:4:1 [9,10]. However, the morphology and oxidation kinetics change when alloying elements are added to such steels. As reported by Asai et al. [11], a small amount of Ni (up to 0.01 wt.%) resulted in a heterogenous scale/steel interface. Moreover, Mouayd et al. [12] revealed that the addition of Si could delay scale growth by forming SiO_2_ at the interface between the steel substrate and the oxide scale. On the contrary, Zhang et al. [13] recently found that increasing the Si content from 0.45 wt.% to 1.8 wt.% caused a significant change in internal oxidation morphology and deepened the internal oxidation depth. These opposite results can be attributed to the simultaneous effect of alloying elements, such as Si, Mn, Ni, and so on. However, in contrast to Si, Cr decreases the oxidation rate of steel [14,15,16,17]. This is because Si promotes the regeneration of Cr_2_O_3_, which is the passivation film formed on the outermost surface of the steel. This film hinders the inward diffusion of oxygen, thereby reducing the oxidation rate. Recently, through the addition of Cr, martensitic stainless steels [18] and press-hardened steels [19,20] with decent oxidation resistance have been developed. However, these studies have all focused on high Cr contents, and there have been relatively few investigations into the high-temperature oxidation of low-alloy steels with Cr contents of 1 wt.% or lower. In addition, high Cr addition increases costs and is detrimental to welding performance [21,22,23]. Hence, determining the optimal Cr concentration is of significance for steels. Up until now, the literature on the high-temperature oxidation behavior of varying Cr-alloyed automotive beam steels has been scarce. Furthermore, eutectic can potentially form at a certain oxidation temperature, leading to a change in the oxidation mechanism and a deterioration in oxidation capacity [24]. Therefore, it is urgent for the effects of varying Cr contents on the oxidation behavior of automotive beam steels to be investigated in depth.

In the present study, the oxidation kinetics of varying Cr-alloyed automotive beam steels were systematically investigated. The morphology evolution of the oxide scale was analyzed using a field-emission scanning electron microscope, an energy-dispersive spectroscope, and X-ray diffraction techniques. The changes in the formation mechanisms of the oxide scale were further examined to understand the oxidation behavior of the investigated steels.

## 2. Materials and Methods

Three varying Cr-alloyed steels were investigated in this study; they were fabricated using a vacuum induction melting furnace. The compositions of the three steels were measured by infrared and optical emission spectrometry, and they are listed in Table 1. After melting, ingots were hot-forged at a homogeneous temperature of 1200 °C. Samples with dimensions of 10 mm × 8 mm × 6 mm were processed from the hot-forged slabs using electrical discharge machining for use in the oxidation experiments. Prior to the experiments, all the specimens were ground with SiC-paper of up to 1200 grade, and were ultrasonically cleaned in ethanol.

Isothermal oxidation experiments were performed in a tubular horizontal furnace without a special atmosphere. The isothermal temperatures used in the experiments were 950, 1050, 1150, and 1250 °C, for durations of 7.5, 15, 30, 60, and 120 min. The samples were introduced into the furnace once the experiment temperature was reached. The weight gain of each sample was measured using a balance with an accuracy of 0.1 mg. Each measurement result under given heat treatment conditions was determined by averaging the results of at least three experiments, and the standard deviation of the experimental values was within ±0.04 mg/mm^2^. After the oxidation experiments, the samples were cold-mounted for microstructure observation.

A field-emission scanning electron microscope was used to carefully observe the surface and cross-sectional states of the oxidized samples at an accelerating voltage of 20 kV, and the elemental distribution of the samples was analyzed by energy-dispersive spectroscopy (EDS). The samples used for observation were revealed with an ethanol solution of 1% HCl. An X-ray diffractometer (XRD) was further utilized to supplementarily reveal the oxide phases of the copper target materials.

## 3. Results

### 3.1. Oxidation Behavior

**Figure 1:**
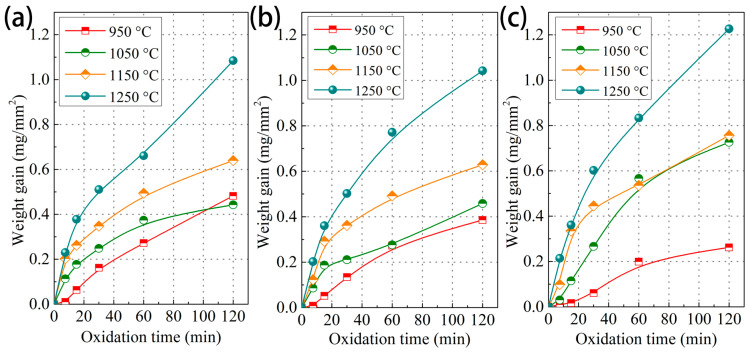
The oxidation weight gain curves of the investigated steels after isothermal oxidation at various temperatures for 120 min. (**a**) Cr0.015 steel, (**b**) Cr0.15 steel, and (**c**) Cr1 steel.

Figure 1 shows the oxidation weight gain curves of the Cr0.015, Cr0.15, and Cr1 steels exposed to 950, 1050, 1150, and 1250 °C for 120 min. All the steels display increasing oxidation weight gain with increasing temperature and time. In particular, in Figure 1a, the oxidation weight gain of the Cr0.015 steel steadily increases with increasing temperature, and under a duration of 120 min, the steel exhibits weight gain values of 0.48 mg/mm^2^ at 950 °C, 0.44 mg/mm^2^ at 1050 °C, 0.64 mg/mm^2^ at 1150 °C, and 1.08 mg/mm^2^ at 1250 °C, respectively. When the Cr content increases to 0.15 wt.% (Figure 1b), the steel exhibits a decreasing trend of oxidation weight gain in comparison with the Cr0.015 case; for example, an oxidation weight gain of 0.39 mg/mm^2^ at 950 °C is achieved (0.48 mg/mm^2^ for Cr0.015 steel). With a further increase in Cr concentration (Figure 1c), the weight gain of the Cr1 steel shows an obvious decreasing value of 0.26 mg/mm^2^ at 950 °C, while it exhibits significantly increased values at 1050, 1150, and 1250 °C. In particular, at 1250 °C, the oxidation weight gain of the Cr1 steel is 1.23 mg/mm^2^, which is even higher than that of the Cr0.015 steel under the same oxidation conditions.

The oxidation behavior could be quantitively described using the oxidation rate constant (*K_p_*); it can be calculated using Equation (1) [25]:

(1) $$∆W_{g}=K_{p}\times t^{n}$$

where Δ*W_g_* is the weight gain per unit area, in mg/mm^2^; *t* is the oxidation time, in min; *n* is the exponent; and *K_p_* is the oxidation rate constant. Simultaneously, take the logarithm on both sides of Equation (1):

(2) $$\ln\left({∆W}_{g}\right)=\ln\left(K_{p}\right)\times n\ln t$$

The oxidation rate constants at different oxidation temperatures were calculated by performing a linear fit using Equation (2), as summarized in Table 2. From the calculated results, the same trend is revealed: the oxidation rate constant decreases at all the isothermal temperatures when the Cr content increases from 0.015 wt.% to 0.15 wt.%. With the Cr concentration increasing to 1 wt.%, a declining oxidation rate constant is obtained at 950 °C; meanwhile, at 1050, 1150, and 1250 °C, the oxidation rate constants of the Cr1 steel are higher than that of the Cr0.015 and Cr0.15 steels.

**Figure 2:**
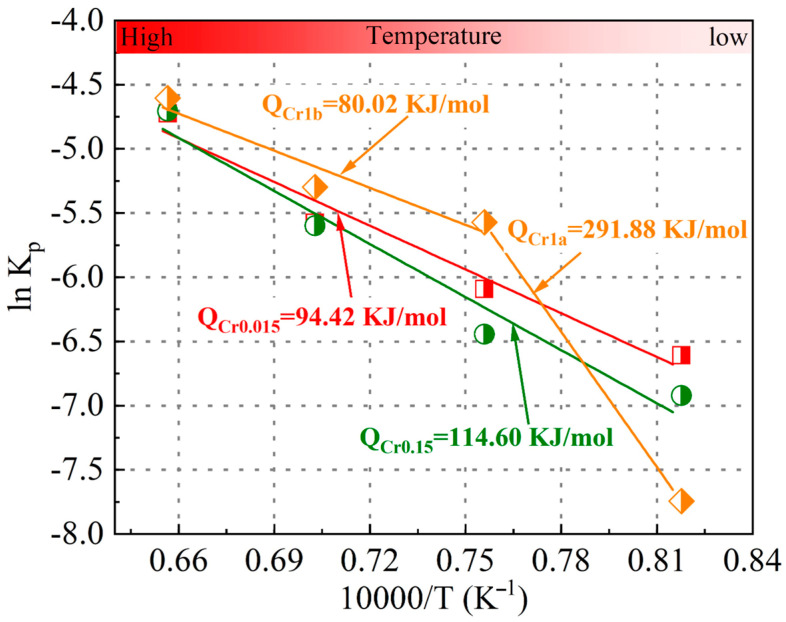
The relationship between ln*K_p_* and 10,000/T of the investigated steels.

The oxidation rate constant (*K_p_*) has an Arrhenius-type relationship with the activation energy (*Q*), as shown in Equation (3) [24,25].

(3) $$K_{p}=K_{0}\exp\left(-\frac{10000\times Q}{RT}\right)$$

where *K*_0_ is the model constant, *R* is the gas constant, and *T* is the absolute temperature. Based on Equation (3), the activation energy can be determined using the slope relationship between ln*K_p_* and 10,000/T, as shown in Figure 2. The determined activation energy values of the Cr0.015 and Cr0.15 steels were 94.42 and 114.60 KJ/mol, respectively. However, two activation energy values were obtained for the Cr1 steel: one was 291.88 KJ/mol under an oxidation temperature of 950 °C; the other was 80.02 KJ/mol when the oxidation temperature varied from 1050 °C to 1250 °C. The obtained activation energy is similar to that of low-alloyed steels, whose activation energy is 73.1–339 KJ/mol [26,27,28]. The two-stage change in the oxidation activation energy of the Cr1 steel corresponds to the change in the oxidation rate constant and weight gain.

### 3.2. Oxide Morphology

**Figure 3:**
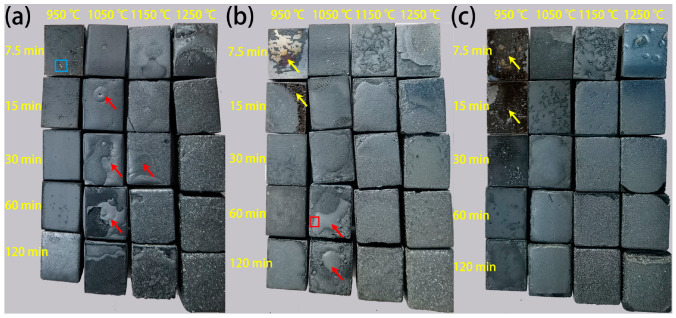
Surface images of the investigated steel after isothermal oxidation. (**a**) Cr0.015 steel, (**b**) Cr0.15 steel, and (**c**) Cr1 steel. The red arrows represent blisters at the surface, and the yellow arrows represent the area of dark oxides.

Figure 3 provides surface images of the investigated steels after oxidation under various temperatures and durations. It can be seen from Figure 3a that the color of the Cr0.015 steel at the initial oxidation stage is dark gray, as exemplified by the sample oxidized at 950 °C for 7.5 min. With increasing isothermal temperature and time, the color gradually brightens, as shown in the surface images of the sample oxidized at 950 °C for 120 min. When the oxidation temperature and time further increase, a rough surface with some covered particles is observed (oxidized at 1150 °C for 15 min and above). The same trend also occurs in the Cr0.15 steel (Figure 3b) and the Cr1 steel (Figure 3c). An apparent difference is that compared to the Cr0.015 steel, the colors of the Cr0.15 and Cr1 steels are much darker at the initial oxidation stage, as indicated by the yellow arrows in Figure 3b,c. Moreover, the occurrence of a rough surface with covered particles is observed at much higher isothermal temperatures and durations, namely, at 1150 °C maintained for 30 min for Cr0.15 steel, and at 1150 °C maintained for 60 min for Cr1 steel. In addition, blisters at the scale surface are formed, as marked using red arrows, and this blistering behavior is relieved with an increasing Cr content, with no obvious blisters at the surface occurring in the Cr1 steel.

**Figure 4:**
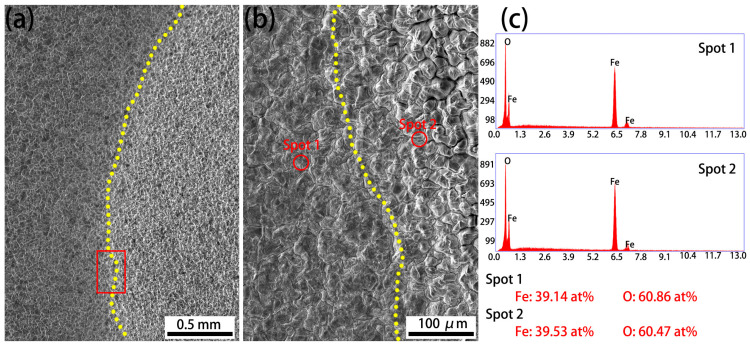
SEM images and EDS profiles of surface morphologies in the blister region of the Cr0.15 steel oxidized at 1050 °C for a duration of 60 min: (**a**) low-magnification image; (**b**) high-magnification image of the red box area in (**a**); and (**c**) EDS profiles of red spot1 and spot2 in (**b**).

The SEM images and the corresponding energy-dispersive spectrometer (EDS) analysis profiles of the red-rectangle region in Figure 3b, showing blisters, are provided in Figure 4. Differences in the grain size of the oxide scale between the blistering region and the flat region, divided by the yellow line, can be observed in Figure 4a,b. The EDS profiles of both regions, however, reveal the same composition, indicating that the oxide scale is Fe_2_O_3_. It is reported that two mechanisms—generated stress and gas (CO/CO_2_) release—are responsible for the occurrence of blistering during the oxidation of the steels [29,30,31]. Figure 4 clearly reveals differences in the grain size of Fe_2_O_3_, indicating internal stress generation during scale formation. The release of gas (CO/CO_2_) is believed to have been limited because the carbon concentration of the investigated steels is quite low [29,30]. Therefore, blistering at the oxide surface occurred mainly due to the internal stress caused by grain size differences.

**Figure 5:**
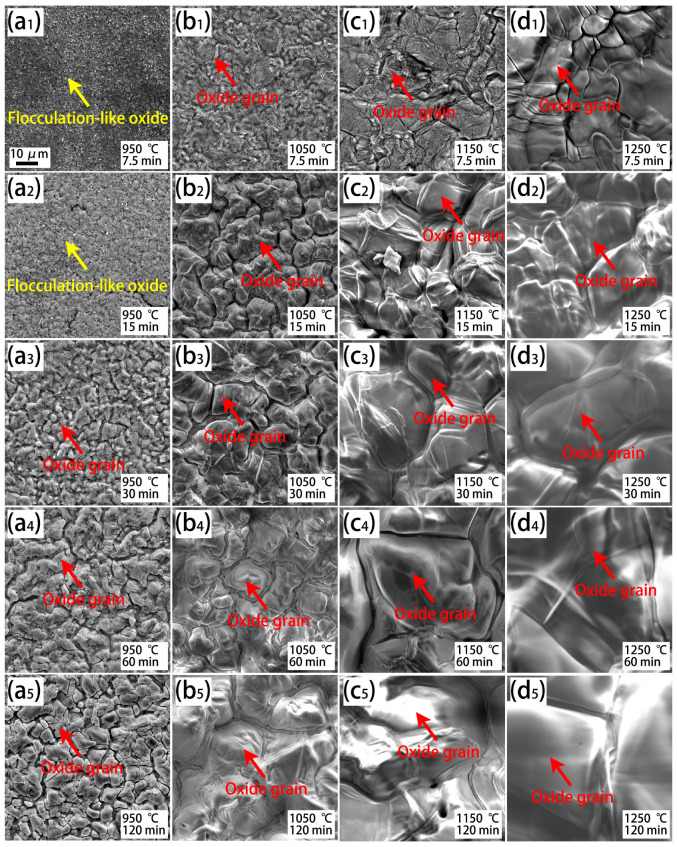
SEM observation of the surface morphologies of the oxide scale on the Cr0.015 steel after isothermal oxidation at various temperatures and durations. (**a1**–**a5**) Exposed to 950 °C for 7.5 min–120 min; (**b1**–**b5**) exposed to 1050 °C for 7.5 min–120 min; (**c1**–**c5**) exposed to 1150 °C for 7.5 min–120 min; and (**d1**–**d5**) exposed to 1250 °C for 7.5 min–120 min.

Figure 5 shows the surface morphologies of the oxide scale for the Cr0.015 steel after isothermal oxidation at various temperatures and durations. Flocculation-like oxides occur under low isothermal temperatures applied for a short duration (Figure 5(a1,a2)). With increasing temperature or duration, the grain boundary of oxides gradually becomes clear and can be identified, for example, in Figure 5(b2). With a further increase in temperature or duration, the oxide grain becomes coarse (Figure 5(b3–d5)). Considering that the morphology of the grain-like oxide in Figure 5(b3) is the same as that in Figure 4, it can be concluded that the oxide grain is Fe_2_O_3_ [32,33].

**Figure 6:**
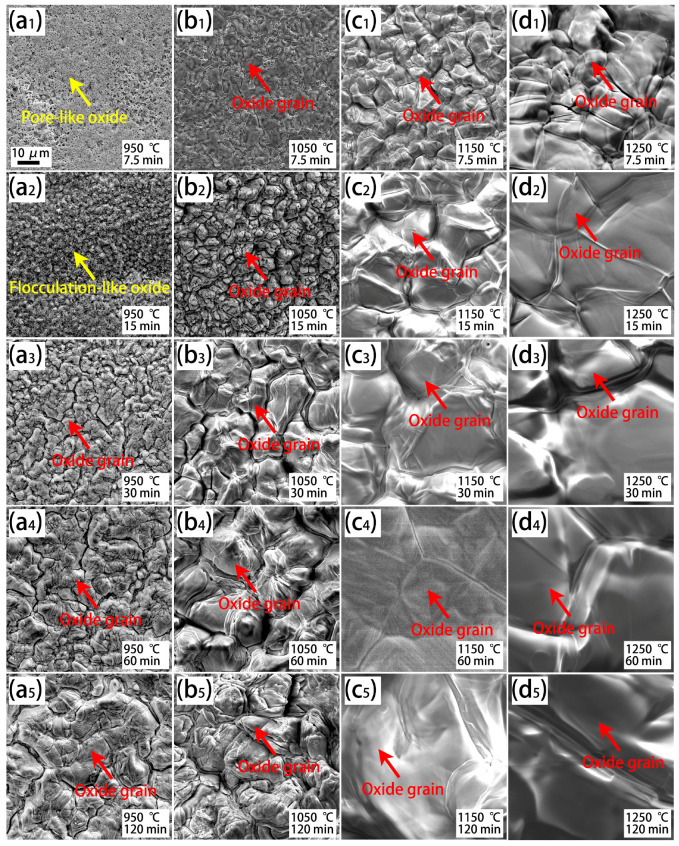
SEM observation of the surface morphologies of the oxide scale on the Cr0.15 steel after isothermal oxidation at various temperatures and durations. (**a1**–**a5**) Exposed to 950 °C for 7.5 min–120 min; (**b1**–**b5**) exposed to 1050 °C for 7.5 min–120 min; (**c1**–**c5**) exposed to 1150 °C for 7.5 min–120 min; and (**d1**–**d5**) exposed to 1250 °C for 7.5 min–120 min.

The surface morphologies of the oxide scale on the steel with a higher Cr content (Cr0.15) after isothermal oxidation are shown in Figure 6. Like the Cr0.015 steel, oxide grains and their growth occur under high isothermal temperatures and long holding times (Figure 6(a3–d5)). The grain-like oxide in Figure 6, with the same morphologies as the oxide in Figure 4, is Fe_2_O_3_, according to the EDS profiles of oxides in Figure 4. Under low isothermal temperatures and short durations (Figure 6(a1,a2)), not only a flocculation-like oxide, but also a pore-like oxide is observed. As shown in Figure 1 and Figure 3, an increase in Cr content can inhibit the oxidation process, and thus pore-like oxide should be an early-oxidation product.

**Figure 7:**
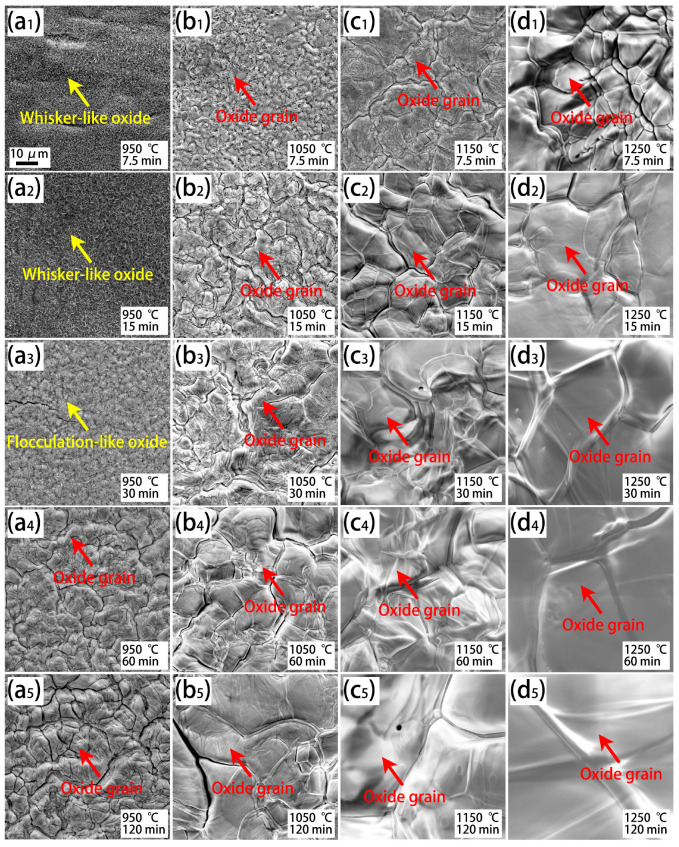
SEM observation of the surface morphologies of the oxide scale on the Cr1 steel after isothermal oxidation at various temperatures and durations. (**a1**–**a5**) Exposed to 950 °C for 7.5 min–120 min; (**b1**–**b5**) exposed to 1050 °C for 7.5 min–120 min; (**c1**–**c5**) exposed to 1150 °C for 7.5 min–120 min; and (**d1**–**d5**) exposed to 1250 °C for 7.5 min–120 min.

Figure 7 illustrates the surface morphologies of the oxide scale on the steel with a further increase in Cr content (Cr1) after isothermal oxidation. Oxide grains and their growth also happen when the Cr1 steel is oxidized at high temperatures maintained for long times (Figure 7(a4–d5)). Under low oxidation temperatures and short holding times, flocculation-like oxide is also observed (Figure 7(a3)). A difference in the oxide morphologies between the Cr1 steel and the other two steels (Cr0.015 and Cr0.15) is that a whisker-like oxide occurs, as shown in Figure 7(a1,a2). Moreover, it can be found that the size of the whisker-like oxide increases with an increase in the holding time from 7.5 min to 15 min. Following 30 min of oxidation, only limited whiskers, but lots of flocculation could be observed, which indicates an evolution in oxide morphology from whisker-like to flocculation-like.

### 3.3. Cross-Sectional Morphology

**Figure 8:**
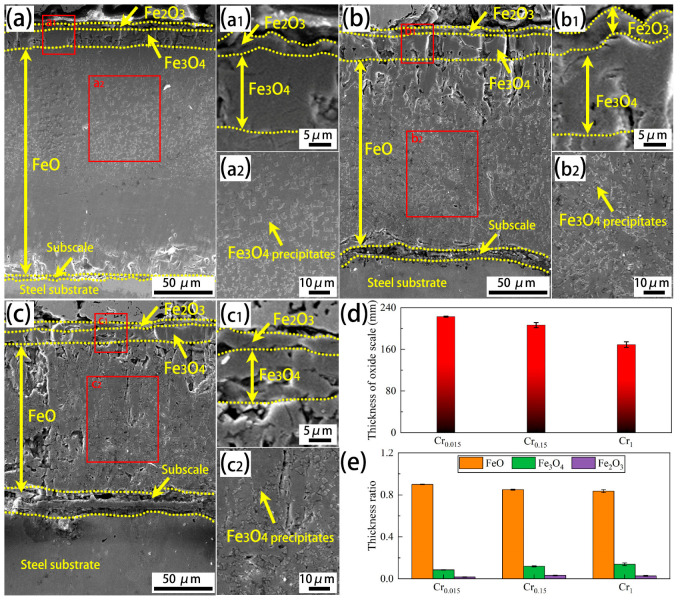
Comparison of cross-sectional morphologies of the oxide scale on the investigated steels after the same isothermal oxidation (held at 950 °C for 60 min): (**a**) Cr0.015 steel; (**b**) Cr0.15 steel; (**c**) Cr1 steel; (**d**) the total thickness of the oxide scale on the investigated steels; (**a**,**e**) the thickness ratio of the different oxide phases on the investigated steels.

**Figure 9:**
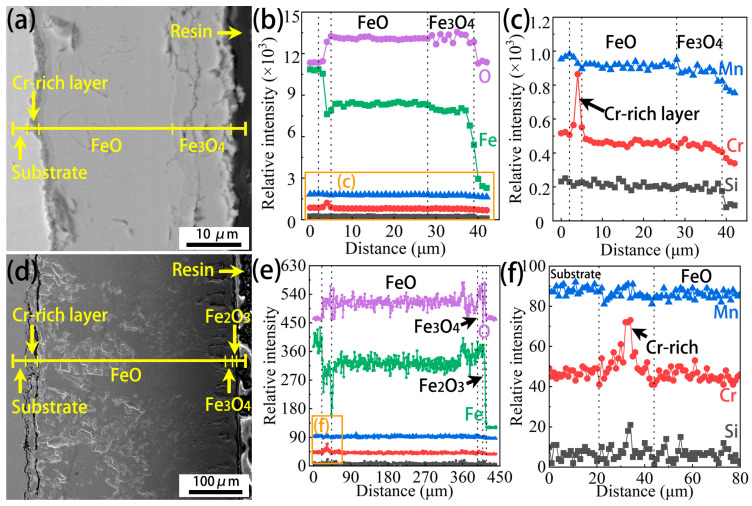
Cross-sectional morphologies and EDS line scan profiles of the Cr0.15 steel oxidized at 1050 °C for 7.5 min (**a**–**c**) and 60 min (**d**–**f**). (**c**) and (**f**) are enlarged figures of the orange-rectangle regions in (**b**) and (**e**), respectively.

**Figure 10:**
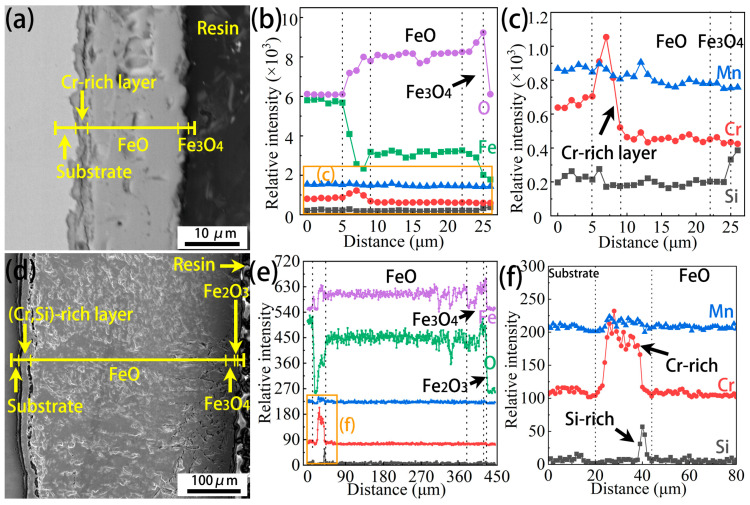
Cross-sectional morphologies and EDS line scan profiles of the Cr1 steel oxidized at 1050 °C for 7.5 min (**a**–**c**) and 60 min (**d**–**f**).

Figure 8 presents the cross-sectional morphology of the oxide scale on the investigated steels. In accordance with the cross-sectional morphology and EDS line scanning, as depicted in Figure 9 and Figure 10, all the studied steels develop a four-layered oxide scale, which, from the outer to the inner layer, consists of an Fe_2_O_3_ layer, an Fe_3_O_4_ layer, an FeO layer, and a subscale on the steel substrate. Based on the EDS results, it can be concluded that the subscale on the steel substrate is a Cr-rich layer or a (Cr, Si)-rich layer. Specifically, for the Cr0.015 steel, the Fe_2_O_3_ layer is thinner than the Fe_3_O_4_ layer, as shown in Figure 8a and its local high-magnification image (Figure 8(a1)). Moreover, many Fe_3_O_4_ precipitates are found in the thick FeO layer, as indicated in Figure 8(a2). The same phenomenon occurs in the Cr0.15 steel (Figure 8(b1,b2)). The difference is that the FeO layer is much thinner for the Cr0.15 steel, while relatively thick Fe_2_O_3_ and Fe_3_O_4_ are observed. When the Cr content increases to 1 wt.%, the thickness of the FeO further reduces (Figure 8(c1,c2)). Based on careful calculation, as shown in Figure 8d, the total thickness of the oxide scale shows a decreasing trend, with the value decreasing from 222.7 mm to 206.4 mm and finally to 169.0 mm, when the Cr content increases from 0.015 wt.% to 0.15 wt.% and finally to 1 wt.%, which correlates well with the decreasing trend of oxidation weight gain versus Cr concentration shown in Figure 1. In addition, the thickness ratio of different oxide phases for the investigated steels (Figure 8e) demonstrates a decreasing thickness ratio of FeO, but a slightly increasing ratio of Fe_3_O_4_ and Fe_2_O_3_. The change in the thickness ratio of the different oxide phases indicates that the decrease in the total thickness of the oxide scale mainly results from the decreasing thickness of the FeO layer.

Cross-sectional morphologies and EDS line scan profiles of the Cr0.15 steel oxidized at 1050 °C for 7.5 min and 60 min are shown in Figure 9. It can be seen from Figure 9a that the FeO and Fe_3_O_4_ layer can be distinguished slightly at the early stage of oxidation. Moreover, between the FeO layer and the substrate, a Cr-rich layer is observed, as illustrated using the corresponding EDS line scan profiles (Figure 9b,c). The total thickness of the oxide scale is about 36 µm at this moment. With increasing oxidation time, the total thickness of the oxide scale increases significantly, up to about 397 µm (Figure 9d). Moreover, a thin Fe_2_O_3_ layer occurs on the Fe_3_O_4_ layer, namely on the surface of the Cr0.15 sample, which aligns with the EDS analysis in Figure 4. Similarly to the results for the early stage of oxidation in Figure 9c, a Cr-rich layer is observed, as shown in Figure 9e,f, but a greater Cr-rich distance is displayed (Figure 9f).

Figure 10 shows the cross-sectional morphologies and EDS line scan profiles of the Cr1 steel oxidized at 1050 °C for 7.5 min and 60 min. The FeO and Fe_3_O_4_ layer, as well as the Cr-rich layer between the FeO layer and the substrate, also occur at the early stage of oxidation (at 1050 °C for 7.5 min), as revealed by the morphologies and EDS line scan profiles in Figure 10a–c. The total thickness of the oxide scale is about 20 µm, which is much thinner in comparison with the Cr0.15 case. An unexpected result is that a much higher total thickness of the oxide scale in the Cr1 steel (411 µm) compared to the Cr0.15 steel (397 µm) occurs when the oxidation time increases to 60 min (Figure 10d,e). From Figure 10f, is can be seen that not only Cr, but also Si, are enriched in the layer between the substrate and the FeO. Moreover, the enrichment degree of Cr is more significant compared with that seen for the Cr0.15 steel, and the enrichment position of Si is close to the FeO layer.

## 4. Discussion

### 4.1. Morphological Evolution of the Oxide Scale

**Figure 11:**
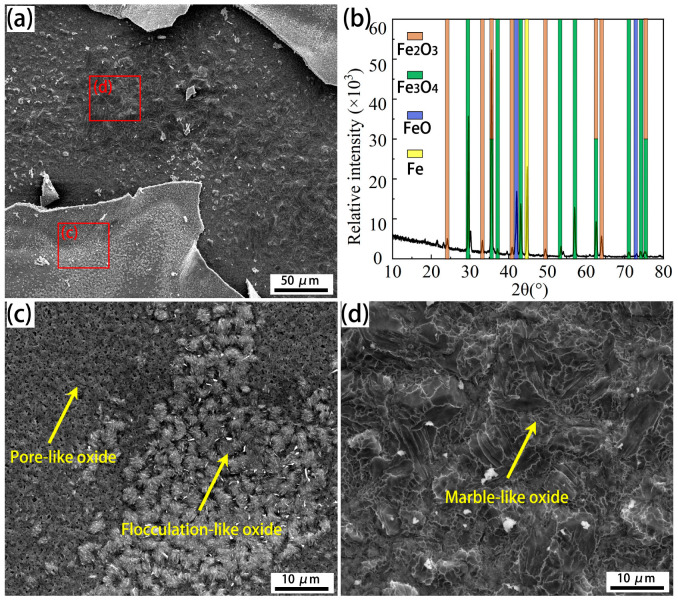
SEM observation of scale morphologies on and below the surface, and the corresponding XRD pattern of the oxide scale for Cr0.015 steel oxidized at 950 °C for 7.5 min. (**a**) Low-magnification image; (**b**) the XRD pattern of scales; (**c**,**d**) high-magnification images of the red regions in Figure 11a.

Varying Cr micro-alloyed steels were designed in this study, and the surface morphological evolution of the oxide scale, especially at the early stage, could be comprehensively understood due to the delayed effect of Cr on morphological evolution. In the steels with higher Cr contents, whisker-like and pore-like oxides were additionally observed at the early stage of isothermal oxidation. With increasing oxidation temperature or isothermal time, a flocculation-like oxide formed, followed by the fine oxide grains, and finally enlarged grains. This whisker-like oxide has been reported in studies on micro-alloyed steel conducted by Liu et al. [28], and their studies indicate that Fe_2_O_3_ could appear with whisker-like and platelet-like morphology, resulting in a dark surface. This is insistent with the surface images in Figure 3c, indicating that the whisker-like oxide is Fe_2_O_3_. Since the whisker-like oxide is not the initial oxide product (the Fe_2_O_3_ layer forms later than the FeO and Fe_3_O_4_ layers as shown in Figure 8), whisker-like morphology should not be the initial morphology of the oxide scale. The earlier morphology of the oxide scale can be obtained by observing the layer below the surface, as shown in Figure 11. The surface and the layer below the surface illustrate the different morphologies of the oxide scale (Figure 11a), and these oxides include Fe_2_O_3_, Fe_3_O_4_, and FeO, as verified by the XRD spectrum (Figure 11b). Flocculation-like oxides form on the pore-like oxide (Figure 11c), while marble-like oxides form on the layer below the surface (Figure 11d). Thermodynamic analysis at 950 °C reveals that the Gibbs energy for the formation of Fe_2_O_3_, Fe_3_O_4_, and FeO is determined to be −340 kJ/mol, −360 kJ/mol, and −370 kJ/mol, respectively [19,34,35,36]. Under the experimental conditions (non-vacuum, without a special atmosphere), the oxygen partial pressure was sufficient for oxide formation, making the Gibbs energy the determining factor for the iron oxide formation sequence. According to the calculation results of the Gibbs energy for the formation of Fe_2_O_3_, Fe_3_O_4_, and FeO, FeO has the lowest Gibbs energy, and thus forms close to the substrate (the layer below the surface). Therefore, the marble-like oxide is FeO, as confirmed by the XRD profile. A flocculation-like oxide and a small amount of whisker-like oxide are observed (Figure 11c), and the flocculation-like oxide occurs later than the whisker-like oxide (Figure 6(a2,a3)), which indicates that flocculation-like morphology is another characteristic of Fe_2_O_3_. The pore-like oxide that forms after FeO, but before Fe_2_O_3_, is Fe_3_O_4_, because the Gibbs energy for forming Fe_3_O_4_ is higher than that for FeO, but lower than that for Fe_2_O_3_ [34,35]. In addition, the peaks in the XRD profile also imply the occurrence of Fe_3_O_4_.

**Figure 12:**
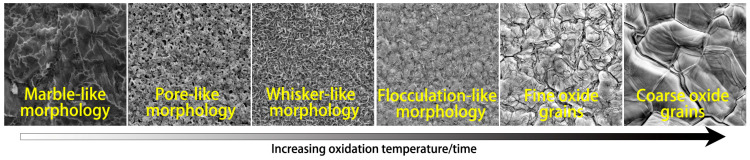
Morphological evolution of the oxide scale.

On the basis of the morphology of the surface and below the surface of the designed varying Cr micro-alloyed steels, the morphological evolution of the oxide scale is summarized and shown in Figure 12. With increasing oxidation temperature or time, the morphology evolution of the oxide scale is as follows: marble-like → pore-like → whisker-like → flocculation-like → fine oxide grains → coarse oxide grains.

### 4.2. Oxidation Kinetics and the Formation Mechanisms of the Oxide Scale

As shown in Section 3.1, increasing the Cr content from 0.015 wt.% to 0.15 wt.% resulted in a decrease in oxidation weight gain, a smaller oxidation rate constant, and a lower oxidation activation energy. However, in the case of 1 wt.% Cr steel, when the temperature was higher than 1050 °C, the opposite results were obtained. It has been reported by Kwon et al. [37] that Cr can increase the resistance of oxidation; however, higher oxidation kinetics with a temperature above 1050 °C was demonstrated in this study when the Cr concentration was increased to 1 wt.%. The aforementioned results indicate that there are changes in the formation mechanisms of the oxides.

**Figure 13:**
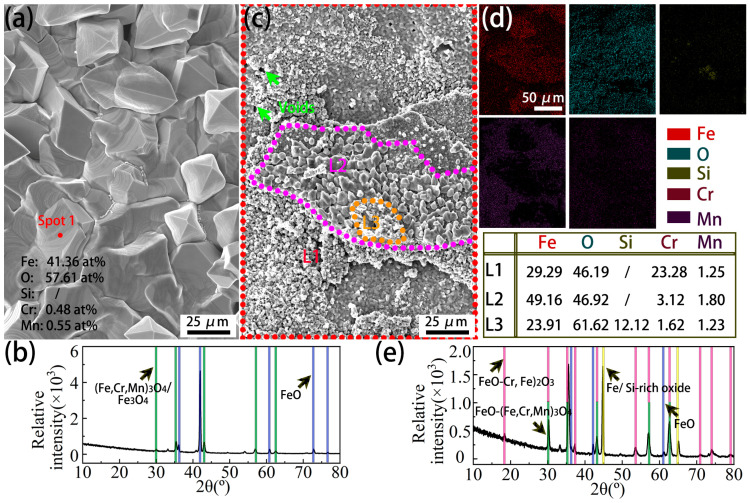
The morphologies of the element-rich layer on both steels oxidized at 1050 °C for 60 min and the corresponding XRD profiles: (**a**) morphologies and (**b**) XRD profiles for the element-rich layer on Cr0.15 steel; (**c**) morphologies, (**d**) the corresponding element distribution obtained through surface scanning of the Cr1 steel, and (**e**) XRD profiles for the element-rich layer of the Cr1 steel.

The cross-sectional morphologies of the Cr0.15 steel and Cr1 steel (Figure 9 and Figure 10) show the occurrence of a Cr-rich layer at the early stage (7.5 min). However, with an increase in the oxidation time to 60 min, the Cr1 steel has a (Cr, Si)-rich layer, in contrast to the Cr-rich layer on the Cr0.15 steel. The morphologies of the element-rich layer on both steels oxidized at 1050 °C for 60 min are further observable in Figure 13. The element-rich layer on the Cr0.15 steel was determined to be mainly (Fe, Cr, Mn)_3_O_4_ using EDS elemental analysis and the XRD profile (Figure 13a,b). It grows beside the FeO layer, as revealed in Figure 9, and thus diffraction peaks of FeO occur as well (Figure 13b). Compared with the Cr0.15 steel, the Cr1 steel displays a complicated element-rich layer, including three sublayers of L1, L2, and L3, as shown in Figure 13c and the corresponding EDS mapping (Figure 13d). Combining the EDS elemental analysis and the XRD profile, it was determined that the sublayers of L1 and L2 are FeO-(Cr, Fe)_2_O_3_ eutectic and FeO-(Cr, Fe, Mn)_3_O_4_ eutectic, respectively. The chemical formula of the L3 sublayer could not be reasonably determined, but it is a Si-rich oxide. The L3 sublayer grows above the L1 and L2 sublayers, and is close to the FeO layer, correlating well with the results in Figure 10, in which the enrichment position of Si is close to the FeO layer. The determined phase composition of the oxides shows that FeO-(Cr, Fe, Mn)_3_O_4_ eutectic forms on the Cr0.15 steel, but on the Cr1 steel, FeO-(Cr, Fe)_2_O_3_ eutectic + Si-rich oxides occur. It has been reported [38,39] that Cr_2_O_3_ will transform into gaseous Cr_2_O_3_ when specimens are isothermally oxidated at a temperature above 1000 °C. In the current study, FeO-(Cr, Fe)_2_O_3_ eutectic formed in L1, and a certain amount of gaseous Cr-rich oxide occurred during the oxidation process, indicated by voids. The formation of FeO-(Cr, Fe)_2_O_3_ eutectic and gaseous Cr-rich oxide results in the inability of the Cr element to prevent the diffusion of Fe cations and O^2−^.

**Figure 14:**
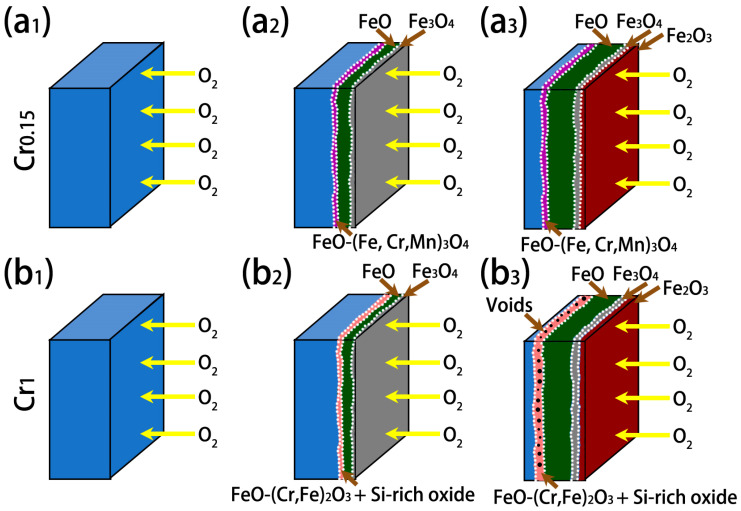
A schematic diagram showing the formation mechanisms of oxides: the temporal evolution of surface oxides on Cr0.15 steel (**a1**–**a3**), and Cr1 steel (**b1**–**b3**) with increasing exposure time.

A schematic diagram showing the formation mechanisms of the oxides on the investigated steels is finally summarized and provided in Figure 14. At the initial stage of oxidation, the O_2_ diffuses to the substrate of the Cr0.15 and Cr1 steels, as shown in Figure 14(a1,b1). With the increasing oxidation time, FeO and Fe_3_O_4_ layers form due to the inward diffusion of O_2_. Meanwhile, FeO-(Cr, Fe, Mn)_3_O_4_ eutectic forms on the Cr0.15 steel, but FeO-(Cr, Fe)_2_O_3_ eutectic + Si-rich oxides occur on the Cr1 steel, due to its much higher Cr concentration (Figure 14(a2–b2)). The relatively thick (Cr, Si)-rich layer, namely FeO-(Cr, Fe)_2_O_3_ eutectic + Si-rich oxides, on the Cr1 steel decelerates the inward diffusion of O_2_, and thus decreases the oxidation rate [40,41,42]. Afterward, a continuous Fe_2_O_3_ layer forms on the pore-like Fe_3_O_4_ layer, with the rough surface providing nucleation sites (Figure 14(a3–b3)). Additionally, when the temperature of the Cr1 steel exceeds 1050 °C, voids form in the (Cr, Si)-rich layer. The occurrence of voids in the (Cr, Si)-rich layer and cavities in the FeO and Fe_3_O_4_ layers leads to the inability to prevent the inward diffusion of O_2_ [24,27,42]. As a result, an increase in the oxidation weight gain and oxidation rate occur in the Cr1 steel.

## 5. Conclusions

In this work, the oxidation behaviors of different Cr-alloyed automotive beam steels (with 0.015 wt.% Cr, 0.15 wt.% Cr, and 1 wt.% Cr) were investigated. The morphologies of the oxide scales were characterized, and the formation mechanisms of the oxide scales were analyzed using field-emission scanning electron microscopy, energy-dispersive spectroscopy, and X-ray diffraction techniques. It was found that an increase in Cr content can enhance the oxidation resistance of automotive beam steels. However, when the Cr content is too high (1 wt.%), the oxidation resistance will be reduced at high temperatures (up to 1050 °C). The main conclusions that can be drawn are as follows:

(1) An increasing Cr concentration of automotive beam steels from 0.015 wt.% to 0.15 wt.% decreases the oxidation kinetics at all the isothermal temperatures and increases the oxidation activation energy from 94.42 KJ/mol to 114.60 KJ/mol. Compared to the steel with 0.15 wt.% Cr, an increase in Cr addition to 1 wt.% results in decreasing oxidation kinetics at an oxidation temperature of 950 °C, but increasing oxidation kinetics when the isothermal temperature is above 1050 °C. Therefore, considering both oxidation resistance and cost, the optimal Cr concentration in automotive crossbeam steel is 0.15 wt.%.

(2) All the automotive beam steels—with 0.015 wt.% Cr, 0.15 wt.% Cr, and 1 wt.% Cr—exhibit a three-layer oxidation scale: the inner FeO layer, the intermediate Fe_3_O_4_ layer, and the outer Fe_2_O_3_ layer. Increasing the Cr content can decrease the thickness of the FeO layer and the total thickness of the oxidation scale.

(3) The Cr-alloyed automotive beam steels have a morphology evolution from marble-like to pore-like, then whisker-like, flocculation-like, fine oxide grains, and finally coarse oxide grains as oxidation progresses. Grain size differences in the fine oxide grains between the different regions is responsible for the occurrence of blistering at the oxide surface, and the blistering behavior is relieved with increasing Cr content.

(4) A Cr-rich layer and FeO-(Cr, Fe, Mn)_3_O_4_ eutectic form in the oxidation scale on the steel with 0.15 wt.% Cr during the oxidation process, while in the 1 wt.% Cr steel, FeO-(Cr, Fe)_2_O_3_ eutectic and Si-rich oxides, as well as a (Cr, Si)-rich layer, occur. The increasing oxidation kinetics of the 1 wt.% Cr steel at an isothermal temperature above 1050 °C is related to voids in the (Cr, Si)-rich layer.

## Figures and Tables

**Table 1:** The chemical compositions of the investigated automotive beam steels (wt.%).

Materials	C	Si	Mn	Nb	Cr	Fe
Cr0.015	0.06	0.02	0.75	0.016	0.016	Bal.
Cr0.15	0.06	0.02	0.78	0.020	0.170	Bal.
Cr1	0.06	0.02	0.82	0.016	1.002	Bal.

**Table 2:** The calculated oxidation rate constants of the investigated steels at different isothermal oxidation temperatures.

Kp (mg^2^/(mm^−4^·min^−1^))		Isothermal Oxidation Temperature			
		950 °C	1050 °C	1150 °C	1250 °C
Materials	Cr0.015	1.35 × 10^−3^	1.89 ×10^−3^	3.79 × 10^−3^	9.45 × 10^−3^
	Cr0.15	0.987 × 10^−3^	1.59 × 10^−3^	3.70 × 10^−3^	9.03 × 10^−3^
	Cr1	0.434 × 10^−3^	3.80 × 10^−3^	5.00 × 10^−3^	10.0 × 10^−3^

## Data Availability

The data that support the findings of this study are available from the corresponding author upon reasonable request.
